# Inter -observer variability in manual measurement of aortic root dimensions in pediatric patients: benefits of using a semi-automated tool

**DOI:** 10.1186/1532-429X-16-S1-P133

**Published:** 2014-01-16

**Authors:** Ramkumar Krishnamurthy, Shaine A Morris, Amol Pednekar, Rajesh Krishnamurthy

**Affiliations:** 1 Texas Children's Hospital, Houston, Texas, USA; 2 Baylor College of Medicine, Houston, Texas, USA; 3 Philips Healthcare, Houston, Texas, USA

## Background

Accurate measurement of maximal aortic root dimensions is important for informed decision making on the timing/nature of aortic valve replacement surgeries in pediatric patients. Currently, the observer manually measures the following metrics to quantify maximum aortic root dimensions: 1) Cusp to Commisure (Cu-Co), and 2) Cusp to Cusp (Cu-Cu) lengths. This introduces significant inter-observer variability (IOV), especially if a followup study is performed in a different institution than the previous study. Hypothesis: A simple post-processing tool that allows the observer to perform semi-automated measurements using reproducible landmarks will enable accurate quantification of maximal aortic root dimensions as well as decrease IOV, thereby leading to reproducibility of measurements.

## Methods

**Figure 1:**
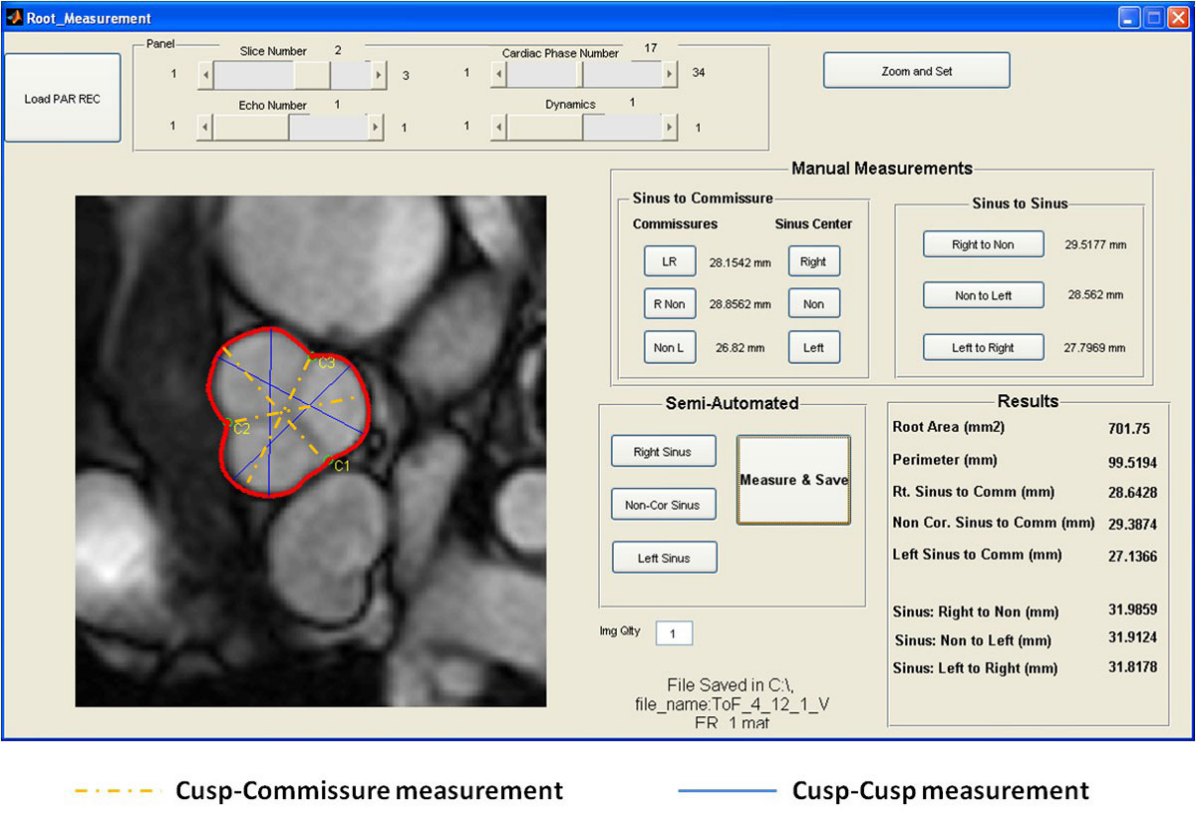
**Tool for accurate and consistent measurement of the aortic root dimensions**. The tool lets the observer perform both manual as well semi-automated measurements. Cusp-commissure and cusp-cusp measurements were performed to identify the maximum dimension of the aortic root. In the semi-automated method, the observers traces easily reproducible landmarks: aortic root circumference and commissure, from which maximum dimensions are automatedly calculated.

An image analysis tool was developed using MatlabTM that can automatically measure the Cu-Cu and Cu-Co lengths from easily reproducible landmarks traced by an observer namely: 1) location of the commissures, and 2) the external margin of the sinuses and aortic circumference (Figure 1). The observer also performed these measurements manually for comparison using the same tool. Study Design: In this retrospective study, cine bSSFP CMR data (temporal resolution: 40 ms, spatial resolution: 1.5*1.5*5 mm) of the aortic root of 11 patients with Tetrology of Fallot (Age: 16.6 ± 7.5 years, LV EF: 59 ± 5.5%) and 15 patients with Marfans' Syndrome (Age: 12.8 ± 8 years, LV EF: 59 ± 6%) were analysed by two experienced observers. Data Processing: Anonymized MR images of the aortic root in the short-axis view were analyzed in early systole and Cu-Co, and Cu-Cu lengths were measured. IOV was computed using Pearson's correlation coefficient (r) and Fisher's z-transformation (z).

## Results

**Figure 2:**
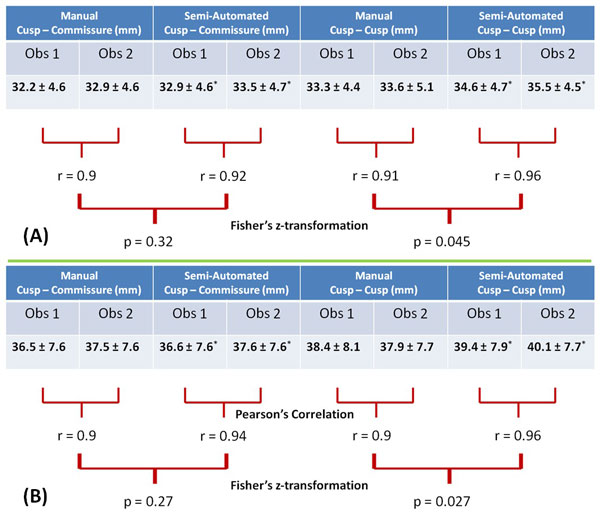
**Aortic Root dimensions measured manually and semi-automatedly are shown in this figure (table) along with the respective Pearson's correlation coefficients (r) and p values for both ToF (A) and MFS patients (B)**. Manual measurements were lower than the semi-automated measurements. A significant improvement in Inter-Observer Variability is seen in semi-automated quantification. This effect is more felt on the cusp-cusp measurements. This can be attributed to the greater consistency seen between observers in tracing easily identifiable landmarks. (Obs - Observer; * - p < 0.001 for paired student's T-test between manual and semi-automated measurements.)

The manual measurements under-estimated aortic root dimensions (Figure 2). The r values (inter-observer agreement) were better for the semi-automated in both ToF and MFS patients. The p-values for z-transformation were a) 0.32 for Cu-Co; 0.045 for Cu-Cu measurements in ToF patients; and b) 0.27 for Cu-Co; 0.027 for Cu-Cu measurements for MFS patients. The p values for Cu-Cu measurements indicate a significant difference between the manula measurements and the semi-automated tool.

## Conclusions

Manual measurements under-estimate aortic root dimensions and present a statistically significant higher IOV when compared to semi-automated measurements. Using an automated tool will reduce the visual subjectivity induced by different observers. We demonstrate that a simple semi-automated tool consistently captures the maximum dimension of the aortic root when compared to the conventional method, and significantly improves IOV. While its benefit might not be substantial in a single center studies, this will be useful in detecting subtle interval change on serial studies conducted at varying imaging centers.

## Funding

None.

